# Gemcitabine and XCT790, an ERRα inverse agonist, display a synergistic anticancer effect in pancreatic cancer

**DOI:** 10.7150/ijms.68404

**Published:** 2022-01-04

**Authors:** Shi-lei Liu, Hai-bin Liang, Zi-yi Yang, Chen Cai, Zi-you Wu, Xiang-song Wu, Ping Dong, Mao-lan Li, Lei Zheng, Wei Gong

**Affiliations:** 1 Department of General Surgery, Xinhua Hospital, Affiliated to Shanghai Jiao Tong University School of Medicine, No. 1665 Kongjiang Road, Shanghai 200092, China; 2 Shanghai Key Laboratory of Biliary Tract Disease Research, No. 1665 Kongjiang Road, Shanghai 200092, China; 3 The Sydney Kimmel Comprehensive Cancer Center, Johns Hopkins University School of Medicine, Baltimore, MD, USA

**Keywords:** Gemcitabine, XCT790, Combination therapy, Pancreatic cancer, Mini-PDX

## Abstract

Pancreatic cancer (PC) is one of the most fatal and chemoresistant malignancies with a poor prognosis. The current therapeutic options for PC have not achieved satisfactory results due to drug resistance. Therefore, it is urgent to develop novel treatment strategies with enhanced efficacy. This study sought to investigate the anticancer effect of gemcitabine and XCT790, an estrogen-related receptor alpha (ERRα) inverse agonist, as monotherapies or in combination for the treatment of PC. Here we demonstrated that the drug combination synergistically suppressed PC cell viability, its proliferative, migratory, invasive, apoptotic activities, and epithelial-to-mesenchymal transition (EMT), and it triggered G0/G1 cell cycle arrest and programmed cell death in vitro. In addition, in vivo assays using xenograft and mini-PDX (patient-derived xenograft) models further confirmed the synergistic antitumor effect between gemcitabine and XCT790 on PC. Mechanistically, gemcitabine and XCT790 suppressed PC by inhibiting ERRα and MEK/ERK signaling pathway. In conclusion, our current study demonstrated for the first time that gemcitabine combined with XCT790 displayed synergistic anticancer activities against PC, suggesting that their combination might be a promising treatment strategy for the therapy of PC.

## Introduction

Globally, PC is a highly aggressive malignant cancer and the seventh leading cause of tumor-associated deaths 1. Operative excision is the only radical treatment for PC, but most sufferers are detected at advanced stages owing to insufficient or vague symptoms in their early stage 2. Chemotherapy represents the main treatment for the patients with advanced stage of PC, and the development in auxiliary chemical treatment have uplifted long-period results for those sufferers 2, 3. Since the FDA accepted the application of gemcitabine in 1996, this medicine has been broadly adopted in the clinical practice as it is the cornerstone and one of the first-line chemotherapy drugs for PC 3, 4. Generally, however, PC responds poorly to most chemotherapeutic agents 5. Moreover, primary and secondary gemcitabine resistance often occurs as PC quickly develops resistance to gemcitabine within weeks of chemotherapy initiation 4, 6, 7. Therefore, it's pivotal to further investigate and unveil the mechanism of gemcitabine resistance, identify the key target genes of chemoresistance, and intervene to strengthen the chemotherapeutic response of gemcitabine in PC. Hopefully, the combination of gemcitabine with targeted inhibition of those key genes can display synergistic antitumor effect.

Emerging evidence suggests that estrogen-related receptor alpha (ERRα) plays a critical role in carcinogenesis and tumor progression through various mechanisms 8-11. In our previous study 10, our team displayed that ERRα was highly expressed in PC patients and promoted the proliferation, migratory and invasive activities of PC cells via MEK/ERK pathway. In addition, inhibition of ERRα could reverse its promoting effects and trigger programmed cell death and cellular cycle arrested in PC cells. Furthermore, recent studies imply that ERRα has been participating in chemoresistance of many tumors, more importantly, targeted inhibition of ERRα could restore chemosensitivity of those cancer cells 11-14. Taken together, these findings indicated that ERRα might be a treatment target for PC.

Based on the above studies, we hypothesized that targeted inhibition of ERRα not only shows anticancer effect but also promotes gemcitabine sensitivity in PC. The present paper demonstrated that combined therapy of gemcitabine and XCT790 to PC cells synergistically suppresses cell viability, as well as proliferative, migratory and invasive activities along with triggering cellular cycle retardation and programmed cell death. Moreover, the anticancer effects of gemcitabine and/or XCT790 were accompanied with downregulation of ERRα and MEK/ERK signaling pathway. These findings indicate that combination treatment of gemcitabine and XCT790 may represent a novel and promising strategy for PC treatment.

## Materials and Methods

### Compounds and cell lines

Gemcitabine and tBHQ were obtained from MedChemExpress, XCT790 and dimethyl sulfoxide (DMSO) were purchased from Sigma-Aldrich. Reagents were subpackaged in 10 μL aliquots for disposable useage and reserved at -20℃ as 1 mmol/L dispersed in 100% DMSO. The concentration of DMSO in vitro was < 0.1% and NC group was subjected to DMSO treatment alone.

Human PC lineage cells (PaTu8988, PANC1 and Mia PaCa-2) and immortalized human noncancerous pancreatic ductal epithelial lineage cell (HPNE) were acquired from the Shanghai Key Laboratory of Biliary Tract Disease Research (PRC). The entire lineage cells were cultivated in DMEM (Gibco) added with 10% FBS (Gibco) and maintained at 37℃ in a 5% CO_2_ moisturized incubating device.

### Cell transfection

ERRα siRNAs and parental NC siRNA were prepared by Genomeditech (PRC). Transfection of siRNAs were performed via RFect reagent (Baidai, China) as per the manufacture's specification. The sense sequence are: si-ERRα-1 (sense, GCGAGAGGAGUAUGUUCUA; antisense, UAGAACAUACUCCUCUCGC); si-ERRα-2 (sense, GAGAGGAGUAUGUUCUACUAA; antisense, UUAGUAGAACAUACUCCUCUC). Lentiviruses expressing full-length sequence of ERRα were established by Genomeditech, empty vector was utilized as control. Cells were subjected to infection by concentrated slow virus at a MOI of 90 for 48 h. Cells were screened by stylomycin (1 μg/ml) for 7 days to establish steady cell transfection and the efficiency of this process was confirmed by qRT-PCR and immunoblotting.

### RNA extraction and quantitation

Overall RNA was abstracted via Trizol reagent (Invitrogen). The reversal transcriptional process was completed via PrimeScript RT reagent kit with gDNA Eraser (Takara). qRT-PCR was conducted via SYBR-Green method (Takara) with a StepOnePlus Real-Time thermocycle (Applied Biosystems). The primers sequences are: ERRα forward, 5'-CACTATGGTGTGGCATCCTG-3' and ERRα reverse, 5'-CGCTTGGTGATCTCACACTC-3'; GAPDH forward, 5'-GGAGCGAGATCCCTCCAAAAT-3' and GAPDH reverse, 5'-GGCTGTTGTCATACTTCTCATGG-3'.

### Western blot assay

Protein abstraction and immunoblotting was completed as above mentioned 13. All antibodies were acquired from Cell Signaling Technology. GAPDH, β-Actin or β-Tubulin were used as internal control.

### Cell viability and growth analysis

Cellular viability and growth were identified via CCK-8 analysis. 3 × 10^3^ cells were inoculated into the 96-well plates with 100 μL complete medium and incubated nightlong. Cells were then subjected to gemcitabine or XCT790 treatment at varying concentrations for 24, 48 and 72 h. 10μL CCK-8 was supplemented to every well and cultivated for additional 2 hours in the dark. The optical density (OD) was determined at 450 nm via a micro-plate reading device.

### Drug combination analysis

Briefly, various combinations of gemcitabine and XCT790 were selected based on the 48 hour half maximal growth inhibition concentration (GI_50_) of each cell line. Synergism between gemcitabine and XCT790 was evaluated via the Chou-Talalay approach and CompuSyn program 15. In a quantitative way, combination index (CI) data describes synergism (CI < 1.0), additivity (1.0 < CI <1.5) and antagonism (CI > 1.5). 48 hour GI_50_ and combination concentrations employed for more analyses are presented by Table 1. In terms of the identification of medicine synergism via certain combination concentrations, score *q* was measured by the fraction product equation of Webb and in a quantitative way, it describes synergism (*q* > 1.0), additivity (*q* = 1.0) and antagonism (*q* < 1.0)^15^.

### Clonogenic assay

Cells were inoculated into 6-well plates at 1000 cells/well, enabled to attach overnight and exposed to DMSO, gemcitabine and/or XCT790 for 48 hours. The medium was removed, cells were cleaned with PBS and cultivated with medicine-free intermediary for 10 d. Afterwards, colonies were treated with fixation via 4% PFA for half an hour and dyed via 0.1% gentian violet for 20 min. The pictures of the dyed plates were collected and colonies were calculated.

### Cell cycle and apoptosis assay

Treated cells were collected and subjected to fixation via 70% ethyl alcohol at 4 ℃ nightlong. Cell cycle distribution was detected via Cell Cycle Analysis Kit (Beyotime) as per the supplier's specification. In short, cells were cultivated via RNase A and PI for half an hour at 37 ℃ away from light and subject to the cellular cycle assay by flow cell technique.

The apoptotic rate of cells were examined by Annexin V-FITC programmed cell death Detection kit (Beyotime, PRC) as per the supplier's specification. In brief, cells were harvested after treatment with gemcitabine and XCT790 in single and in combination for 48 hours, and then re-suspended with binding buffering solution, dyed with 5 μL Annexin V-FITC and 10 μL PI for 20 min at RT in the dark. Flow cell technique assay was used to study programmed cell death.

### Transwell migration and invasion assay

Chamber inserts (Corning) and BioCoat Matrigel Invasion chamber inserts (Corning) were used to examine cell migration and invasion capabilities, respectively. 3 × 10^4^ cells with 200 μL sera-free intermediary were supplemented to the upper well, and 700 μL of medium involving 10% FBS was supplemented to the lower well. The cells were incubated for 24 hours and then fixed with 4% PFA for 30 minutes and dyed with 0.1% gentian violet for 20 minutes. Interiors of the inserts were carefully cleaned with PBS and cleaned by wet cotton sticks, cells remained at the bottom were observed and calculated via the microscopic device in five stochastic different fields.

### Wound healing analysis

Cells were inoculated in 6-well plates at a great density and cultured to more than 90% cell confluence. Wound lines across the superficies of plates were scratched with same strength via a 200 μL sterile plastic tip. The cells were cleaned with PBS to remove debris and then cultured in sera-reduced DMEM medium for 24 hours. A microscope was used to monitor the closure of the wound at 0 and 24 hours.

### Xenograft model

BALB/c nude mice (4-week-old, weight 18-22g) were acquired from Shanghai Laboratory Animal Center of CAS. PaTu8988 cells (2 × 10^6^ per mouse) were introduced into the left axilla of nude mice through subcutaneous injection. The next day, the animals were stochastically separated into 4 groups of 5: NC group, gemcitabine group, XCT790 group and gemcitabine + XCT790 group. The mice were injected with gemcitabine (60 mg/kg) and/or XCT790 (2.5 mg/kg) by intraperitoneal (IP) injections 3 times a week for 4 weeks, NC group were subjected to DMSO treatment only. Body weight and tumor volume (1/2 × length × width^2^) of mice were monitored weekly. 8 hours posterior to the final treating, the animals were sacrificed, and the tumors were harvested and weighted for further assays. Animal assays were accepted by the ethical board of Xinhua Hospital Affiliated to SJUSM.

### Immunohistochemical assay

IHC was completed as per the normal steps^16^. The antibody against ERRα was purchased from Abcam, while the rest of antisubstances were obtained from Cell Signaling Technology. The sections were observed via a microscopic device (Leica) and the analysis of expressing level was evaluated by Image J program.

### Mini-PDX models and drug sensitivity analysis

Mini patient derived xenograft (Mini-PDX) models were established as previously described 17. Brief flowchart of mini-PDX assay is shown in Figure 6A. The PC samples were acquired from a patient in the Department of General Surgery, Xinhua Hospital. The patient didn't have chemical treatment or radiation theraphy prior to operative excision, and was later confirmed as ERRα-high tissue by IHC based on our previous scoring system (Figure 6C) 10. The human studies were accepted by the ethical board of Xinhua Hospital Affiliated to SJUSM, and written informed consent was acquired from the staff in the Department of General Surgery, Xinhua Hospital. Briefly, PC tissues were cleaned with HBSS, digested with collagenase, and the cells were collected and transferred to Mini-PDX capsules. Then the capsules were subcutaneously implanted in the subcutaneous tissue of nude mice (4 weeks old, weighted 18-22g). Nude mice were randomized into 4 groups of 5, every animal was given 3 capsules. Gemcitabine (60 mg/kg, IP, day 1 and 5) and/or XCT790 (2.5 mg/kg, IP, day 1, 3 and 5) were used for 7 d, NS was utilized as the control. RFU were detected via the CellTiter-Glo® Luminescent Cell Viability Assay (Promega). Proliferation speed was evaluated via the following formula:

Proliferation rate = (RFU^D7^-RFU^D0^)_drug_/(RFU^D7^-RFU^D0^)_placebo_

### Statistical analyses

All experiments were performed a minimum of 3 times, results were expressed as average ± standard deviations (SD). The numerical results were assayed by two-tailed unpaired Student's *t*-test between 2 groups via GraphPad Prism. *P* scores of < 0.05 had significance on statistics. Half maximal growth inhibition concentration (GI_50_) and CI were computed by CompuSyn program. A CI score < 1 or *q* value > 1 was considered synergism.

## Results

### The combination of gemcitabine and XCT790 exerts synergistic cytotoxicity in PC cell lines

To address the potential of ERRα in the gemcitabine sensitivity of PC cells, our team first knocked out ERRα expression in the PaTu8988 and PANC1 cell lines whereas overexpression ERRα in Mia PaCa-2 cell line (Figure S1A). Then cells were exposed with indicated concentrations of gemcitabine for 48 hours. As shown in Figure S1B, depletion of ERRα strengthened the sensitiveness of PaTu8988 and PANC1 cells to gemcitabine and decreased their gemcitabine GI_50_ values compared with that of the control cells. In contrast, ERRα overexpression enhanced gemcitabine resistance and increased the GI_50_ value in Mia PaCa-2 cells (Figure S1B). Hence, we assumed that ERRα specific inverse agonist XCT790 would sensitize PC cells to gemcitabine, thus the combination of gemcitabine and XCT790 may exert synergistic cytotoxicity in PC cells.

**Figure 1:**
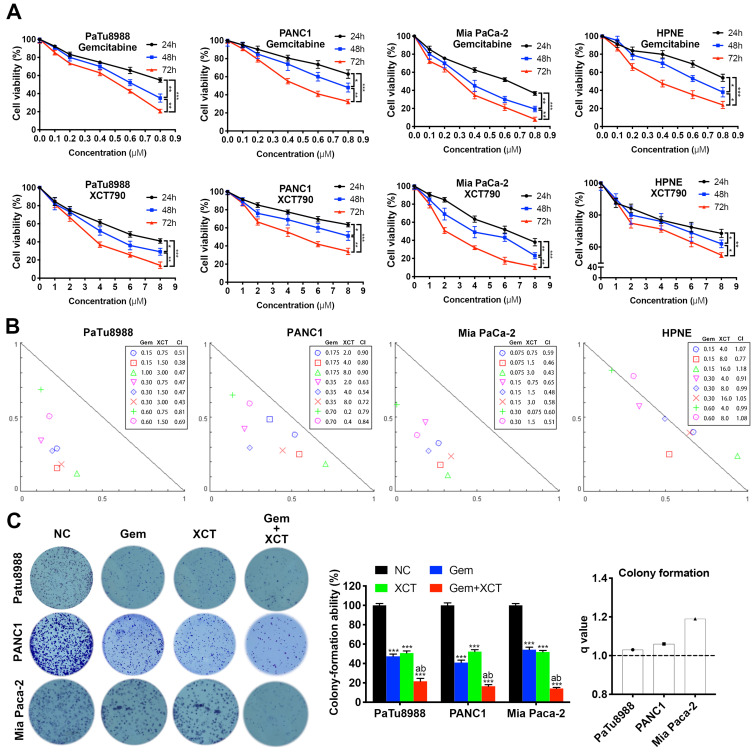
**Gemcitabine and XCT790 exerted synergistic cytotoxicity in PC lineage cells.** A, Gemcitabine and XCT790 single treatment inhibited cell viability and proliferation of PC cell lines and HPNE in a dosage-reliant and time-reliant way. Cells were exposed to a variety of thickness of gemcitabine or XCT790 for 24, 48 or 72 hours, whose activity was detected by CCK-8 analysis. B, The combination of gemcitabine and XCT790 showed synergistic effect in PC cell lines. Gemcitabine and XCT790 were used in combination to treat PC cell lines and HPNE cells at the indicated concentrations for 48 hours. Normalized isobologram was plotted by CompuSyn Software, CI values < 1.0 demonstrated that the combinations are synergistic. C, The combination of gemcitabine and XCT790 showed synergistic inhibition of PC cell colony formation. The data were analyzed using Student's t-tests. All outcomes are expressed as average ± SD of 3 separate assays. CI value < 1 indicates synergism; *q* score is described as bar graph and > 1.0 denotes synergism. *P < 0.05, **P < 0.01, ***P < 0.001 in contrast to the NC group; a and b represent to compare with the GEM or XCT group, *P* ˂ 0.05, respectively.

To address this hypothesis, we performed cell viability assays to evaluate the effects of gemcitabine and XCT790 on PC lineage cells and HPNE. As presented by Figure 1A, either gemcitabine or XCT790 single treatment remarkably suppressed cellular activity via a dosage-reliant and time-reliant way. More importantly, strong synergistic effects were observed in all three PC cell lines after gemcitabine and XCT790 combination treatments (Figure 1B). The GI_50_ values and dosages for combination treatments were presented by Table 1. Afterwards, colony forming activity after treatment of gemcitabine and/or XCT790 was assessed (Figure 1C). The results showed that gemcitabine and XCT790 synergistically decreased the colony formation activity of PC cells.

### Enhanced G0/G1 cell cycle arrest and apoptosis induction by combined treatment of gemcitabine and XCT790

**Figure 2:**
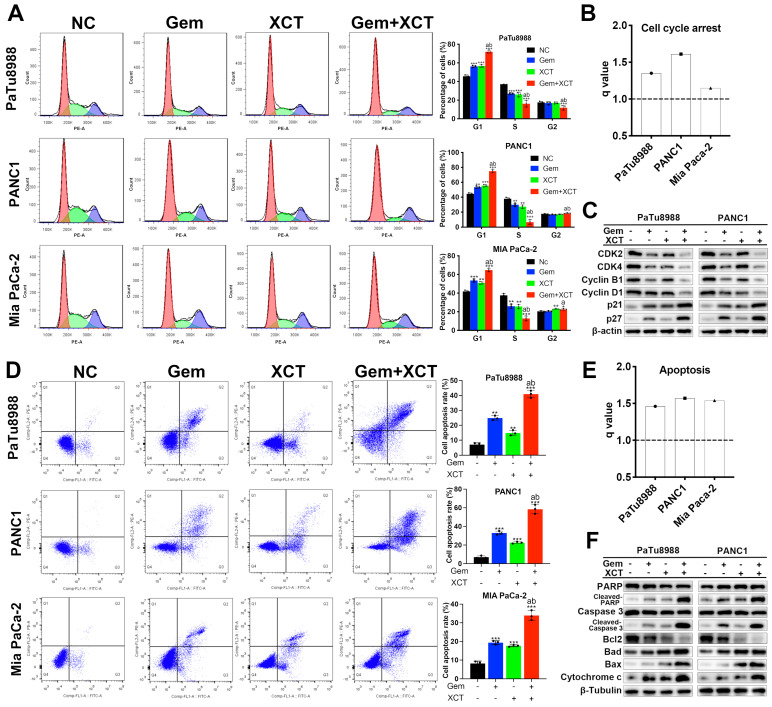
** Gemcitabine and XCT790 synergistically induced G0/G1 cellular cycle retardation and apoptotic activity in PC cells.** A and D, Cellular cycle analysis and apoptosis analysis were completed via flow cell technique. B and E, All q values were > 1.0, suggesting the synergistic effect of gemcitabine + XCT790 on induction of G0/G1 retardation and apoptotic activity in PC cells. C and F, Immunoblotting was applied to identify G0/G1 cellular cycle-related and apoptotic activity-related markers. The data were analyzed using Student's t-tests. The numerical results were expressed as the average ± SD of 3 separate assays.* q* score is described as bar graph and > 1.0 denotes synergism. *P < 0.05, **P < 0.01, ***P < 0.001 in contrast to the NC group; a and b represent to compare with the GEM or XCT group, *P* ˂ 0.05, respectively.

Flow cell technique was adopted to examine the cellular cycle and cell apoptotic rate. The results revealed that gemcitabine and/or XCT790 remarkably elevated the percentage of G0/G1 phase and the level of apoptotic cells (Figure 2A and D), and synergism was observed between gemcitabine and XCT790 (Figure 2B and E). Moreover, cellular cycle-related and apoptotic activity-associated markers were identified by immunoblotting, the results were coherent with the observed G0/G1 cellular cycle retardation and triggering of apoptosis (Figure 2C and F). Among those markers, a significant increase in the ratio of Bax/Bcl-2 was found (Figure S2). These findings suggested that gemcitabine and XCT790 altered the key proteins associated with the modulation of cellular cycle and programmed cell death to confer its growth suppressive potency.

### Gemcitabine and XCT790 synergistically inhibits migratory, invasive and EMT process in PC cells

**Figure 3:**
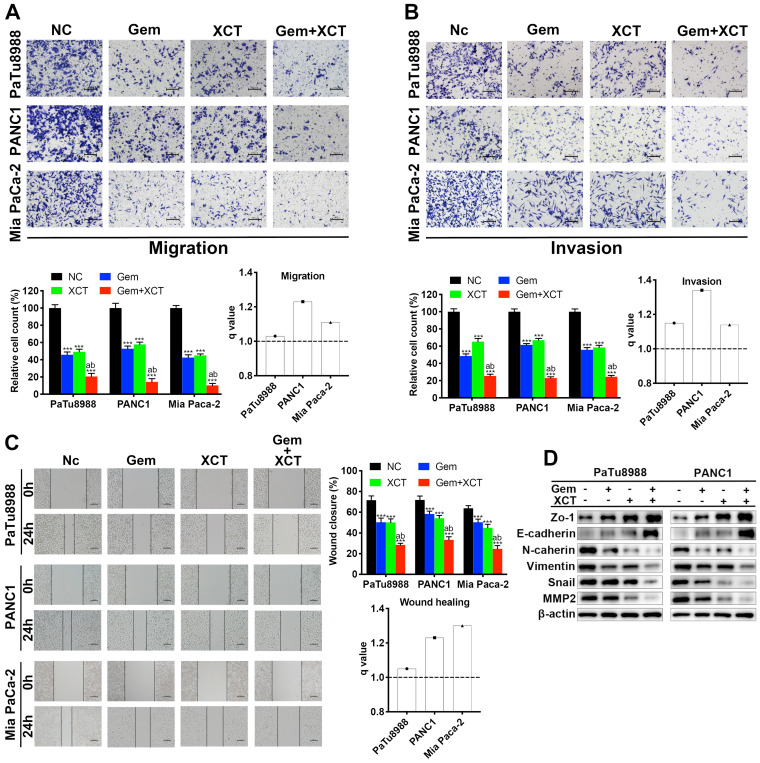
** Gemcitabine and XCT790 suppressed migration, invasion and EMT process synergistically.** (A) Transwell migration analysis and (C) wound healing analysis were carried out to assess the effect of gemcitabine, XCT790 or their combination on PC cells' migratory ability. (B) Transwell invasion assay was conducted to evaluate the PC cells' invasive ability. (D) Western blot analysis was performed to examine epitheliums biomarkers (ZO-1 and E-cadherin) and mesenchyma biomarkers (N-cadherin, vimentin, snail and MMP2) after treatment with gemcitabine and/or XCT790. The data were analyzed using Student's t-tests. *q* score is described as bar graph and > 1.0 denotes synergism. *P < 0.05, **P < 0.01, ***P < 0.001 in contrast to the NC group; a and b represent to compare with the GEM or XCT group, *P* ˂ 0.05, respectively.

As presented by Fig 3A-C, treatment with gemcitabine or XCT790 alone significantly weakened the migration (Figure 3A and C) and invasion (Figure 3B) capacities of PC cells and their combination treatment displayed synergistic effect. It is widely known that EMT plays fundamental effects in metastasis formation and drug resistance of PC 18. Hence, our team studied the expression of EMT-related biomarkers via immunoblotting (Figure 3D). The expressing levels of epithelial markers (ZO-1, E-cadherin) were increased while that of mesenchymal markers (N-cadherin, vimentin, snail and MMP2) were reduced in PC cells after exposed to gemcitabine and/or XCT790. Collectively, these results indicated that gemcitabine and XCT790 inhibited PC cell migratory, invasive and EMT activities in a synergistic manner.

### Gemcitabine and XCT790 display anticancer effect via downregulation of ERRα and MEK/ERK signal path

**Figure 4:**
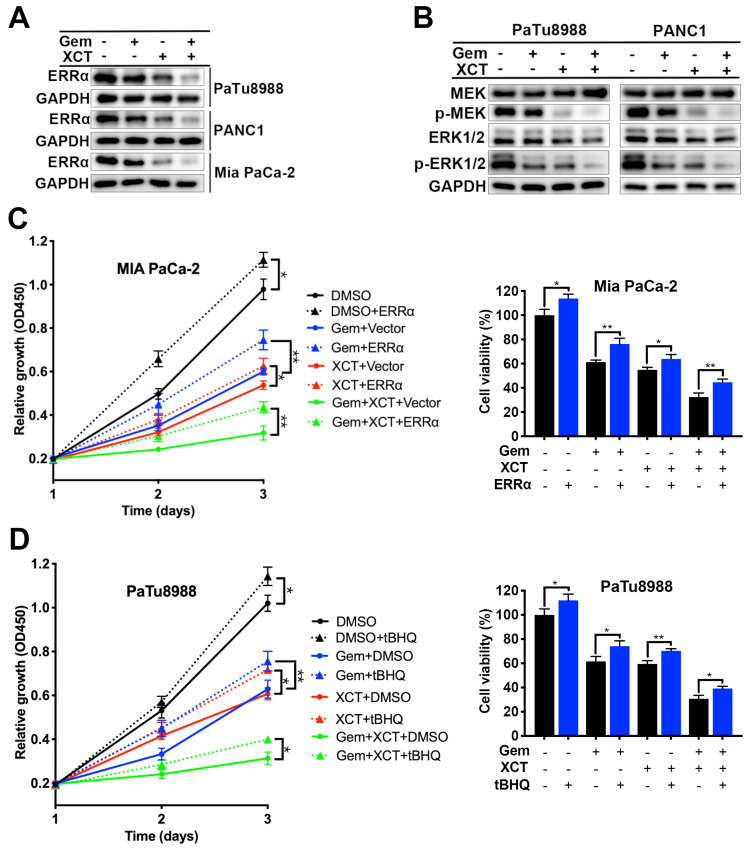
**Gemcitabine and XCT790 triggered synergistic anticancer activity via inhibiting ERRα and MEK/ERK signaling pathway.** A and B, The expressing levels of ERRα and MEK/ERK pathway proteins were identified via immunoblotting. C and D, The cytotoxicity induced by gemcitabine, XCT790 and their combination were significantly attenuated by ERRα overexpression and pre-treatment of ERK activator tBHQ. PC cells were incubated with gemcitabine and/or XCT790 for 2 days after stably overexpressing ERRα or pre-treated with 50 μmmol/L tBHQ for 8 hours. Cellular activity was assessed via CCK-8 analysis. The data were analyzed using Student's t-tests(*P < 0.05, **P < 0.01 and ***P < 0.001).

Our previous research has reported that ERRα facilitates PC development via MEK/ERK signal path 10. Therefore, we sought to investigate whether gemcitabine and XCT790 exhibit anticancer effect by interfering with ERRα and MEK/ERK pathway. Indeed, treatment with gemcitabine and/or XCT790 significantly suppressed protein levels of ERRα, p-MEK and p-ERK1/2, whereas the levels of MEK and ERK were not affected (Fig 4A and B). Rescue experiments by overexpressing ERRα and pre-treatment with ERK activator tBHQ demonstrated that either ERRα overexpression or tBHQ could attenuate the cytotoxicity of gemcitabine and/or XCT790 on PC cells (Figure 4C and D). Generally, those data revealed that ERRα and MEK/ERK signal path facilitated the antitumor effect of gemcitabine and XCT790 on PC cells.

### Gemcitabine and XCT790 synergistically suppress the proliferative activity of PC in vivo

**Figure 5:**
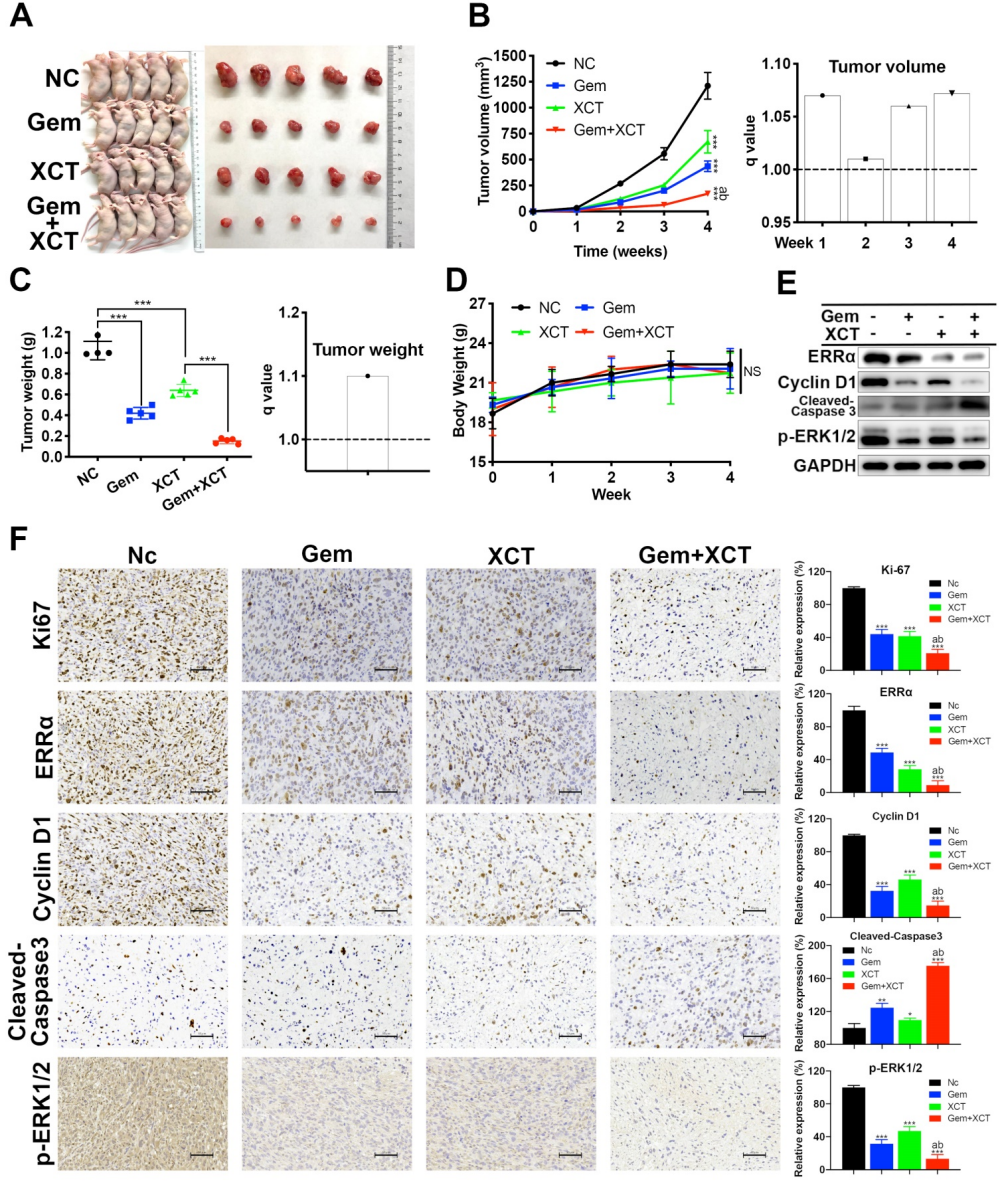
**Gemcitabine and XCT790 potently inhibited in vivo PC tumor growth.** A, Images of the xenograft models and harvested tumors. B and C, Gemcitabine and XCT790 synergistically suppressed tumor growth. D, No significant change of the body weight was observed in all treated mice, suggesting that the gemcitabine and/or XCT790 were well-tolerated. E and F, Western blot analysis and IHC staining were conducted in the indicated xenograft tumors. The data were analyzed using Student's t-tests. The numerical results were expressed as the average ± SD of 3 independently performed assays.* q* score is described as bar graph and > 1.0 denotes synergism. *P < 0.05, **P < 0.01, ***P < 0.001, NS: not significant in contrast to the NC group; a and b represent to compare with the GEM or XCT group, *P* ˂ 0.05, respectively.

We next evaluated the efficacy of gemcitabine and XCT790 in vivo via a heterograft nude murine model treated with PaTu8988 cell transplantation. Nude mice were randomly separated into 4 groups: the NC group, the gemcitabine group, the XCT790 group and the gemcitabine + XCT790 group. Posterior to 4 weeks of treatments, it was discovered that the sizes and weights of the cancers from the gemcitabine-treatment group and XCT790-treatment group were remarkably reduced compared with the NC group (Figure 5A-C). In addition, the combination was more effective and had synergistic effect. There wasn't remarkable difference regarding body weights of the nude mice among the 4 groups, suggesting that the treatments were well-tolerated (Figure 5D). Then the tumors were adopted to immunoblotting and IHC assays. The outcomes showed that gemcitabine and XCT790 single treatment significantly decreased the levels of ERRα, Ki67, cyclin D1 whereas increasing that of cleaved caspase-3, and the combination treatment was related to stronger variation in the proportion of the aforementioned markers (Figure 5E and F). Those outcomes were consistent with the in vitro outcomes, which further verified the antitumor effect and synergism of gemcitabine and XCT790 in PC.

**Figure 6:**
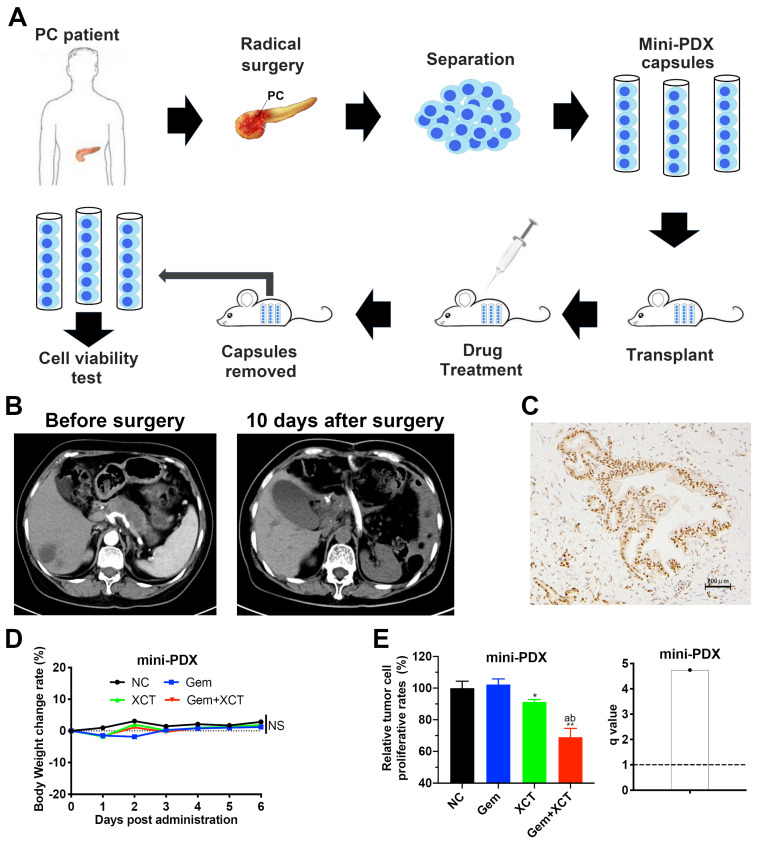
**A novel in vivo drug-response assay, mini-PDX, was performed and demonstrated the synergism between gemcitabine and XCT790 in PC.** A, An overview of the protocol of the generation of mini-PDX pattern. B, Chest scans of the PC patient before and 10 days after surgical resection. C, The PC tissues for mini-PDX were identified as ERRα high-expressing by IHC according to our previous scoring method^10^. D, The body weight of the animals treated with gemcitabine and/or XCT790 didn't exhibit remarkable variations compared with that of mice treated with placebo. E, The combination of gemcitabine and XCT790 exhibited synergistic effect and greater inhibitory effects on mini-PDX models than their single treatment. The data were analyzed using Student's t-tests. *q* score is described as bar graph and > 1.0 denotes synergism. *P < 0.05, NS: not significant in contrast to the NC group.

Mini-PDX has been proved as a rapid and systemic in vivo medicine susceptibility analysis, using patient-derived primary tumor cells, to reliably and precisely assess drug responses of tumor 17, 19-21. We constructed a mini-PDX pattern via fresh primary PC cells from PC tissues by operative excision, in which ERRα was highly expressed (Figure 6B and C). Cohorts of mini-PDX capsules-bearing mice were treated with placebo, gemcitabine, XCT790, or gemcitabine + XCT790 for 7 days. All mice maintained body weight within 5% of initial weight (Figure 6D). Cell viability assays indicated that although no significant effect was observed in gemcitabine treatment group, which was most likely due to the primary resistance to gemcitabine. The combination of gemcitabine and XCT790 remarkably and synergistically decreased the proliferation of tumor cells in mini-PDX models (Figure 6E), suggesting their combination as a better therapeutic strategy than their monotherapy for ERRα-high PC.

## Discussion

In recent years, the incidence of PC is rising globally and it remains a highly aggressive and deadly tumor 22. The therapeutic strategies for PC have not progressed significantly, and gemcitabine-based chemical therapy is still the normal choice for patients with PC for over 20 years 23. Furthermore, the rapid and common development of chemoresistance still causes inferior prognostic results of PC 24. In fact, the 5-year OS for PC has barely improved all these years 25. Thus, it is imperative to determine the key molecular drivers in a precision medicine approach and hopefully better therapeutic options for treating PC.

The orphan acceptor ERRα has been discovered to be related to tumor developmental process, metastasis and chemoresistance 9, 10, 12, 14, 26. Our previous study has pointed out the crucial effect of ERRα in PC development, revealing that ERRα might be a potential diagnosis and treatment target for PC. In this research, we first observed that downregulation of ERRα strengthened the susceptibility of PC cells to gemcitabine, then we explored the possible enhanced effect of gemcitabine with ERRα inverse agonist XCT790. Our in vitro data showed that gemcitabine and XCT induced synergistic cytotoxicity in PC cells, leading to decrease of cellular activity, reduced proliferation, migration and invasion, and triggering of G0/G1 cellular cycle retardation and programmed cell death. In addition, gemcitabine and XCT790 exhibited their anti-tumor efficiency through inhibition of ERRα and MEK/ERK signaling pathway. Coherent with the results of in vitro study, the in vivo study herein also confirmed the synergistic cytotoxicity of gemcitabine and XCT790 in PC. The cancer size and weight of PC cell heterograft patterns were remarkably decreased after treatment with gemcitabine or XCT790, especially with their combination. G0/G1 cell cycle arrest, apoptosis, and downregulation of ERRα and MEK/ERK pathway were also confirmed by immunoblotting and IHC analysis via xenograft tumors. Moreover, mini-PDX models were used in PC for the first time to assess ERRα-high PC patient-derived primary cells responses to gemcitabine and/or XCT790. The results indicated the synergistic effect of gemcitabine and XCT790. However, single treatment of gemcitabine showed no significant effect on mini-PDX models, which may due to the primary resistance to gemcitabine. Hence, more verification from researches with bigger specimen scale is required. In general, these findings suggested that the combination of gemcitabine and XCT790 may represent a promising treatment strategy for PC with high ERRα expression.

Regulation of the cell cycle delicately controls the balance of several concerted processes whose dysregulation is involved in triggering apoptosis and is a hallmark of tumor proliferation and drug resistance 27, 28. The antitumor effect of gemcitabine is primarily reliant on inhibition of DNA synthesis, leading to proliferative activity inhibition, cell cycle arrest and apoptosis. In addition, gemcitabine resistance may result from mitigation of gemcitabine-induced cell cycle arrest and apoptosis 4, 29, 30.Therefore, chemotherapeutic agents that can induce and enhance the potency of gemcitabine on cellular cycle arrest and apoptotic activity are of great promise. Our outcomes revealed that gemcitabine and in a coordinated way, XCT790 elevated the percentage of G0/G1 phase and apoptotic cells by flow cytometry. Further study demonstrated that gemcitabine and XCT790 could downregulate the expression level of CDK2, CDK4, cyclin B1 and cyclin D1, whereas increased that of p21 and p27. Meanwhile, the apoptosis induced by gemcitabine and/or XCT790 was accompanied by downregulation of Bcl-2 and upregulation of cleavage-PARP, -caspase 3, Bad, Bax, histohematin c and especially the rate of Bax/Bcl-2. Taken together, those data indicated that gemcitabine and XCT790 could disrupt PC cell cycle progression and cause apoptosis in a synergistic manner.

PC is extremely aggressive and associated with a poor prognosis because it is prone to distant metastasis 31. EMT is generally accompanied by phenotype variation in oncocytes favoring a mesenchyme cell phenotype with greater aggression, and considered as a major driver of tumor progression from initiation to metastasis 24, 32, 33. Emerging evidence suggests that EMT represents a key step in the regional development and metastatic activity of PC 34. Moreover, EMT process is closely related to chemoresistance in PC, inhibition of which could result in enhancing sensitivity to gemcitabine therapy 18, 24, 35. Our present study showed that gemcitabine and XCT790 dramatically inhibited the migratory and invasive capability of PC cells in a synergistic manner. Moreover, treatments with gemcitabine and/or XCT790 resulted in an increase of epithelial markers and a decrease of mesenchymal markers, which indicated the inhibition and reversal of EMT process. All these data highlighted the potential benefits of combination therapy with gemcitabine and XCT790 in preventing metastasis from PC.

Given that MEK/ERK pathway plays a crucial role in the survival, development, gemcitabine resistance and ERRα-promoted progression of PC 10, 36, 37, we explored whether it is involved in the effectiveness of gemcitabine and XCT790 in PC. As expected, gemcitabine and/or XCT790 dramatically decreased phosphorylation of MEK and ERK1/2, meanwhile, the cytotoxicity effect of gemcitabine and XCT790 could be attenuated by ERRα overexpression and tBHQ (ERK activator). These findings revealed that gemcitabine and XCT790 displayed antitumor effect in PC through suppression of ERRα and MEK/ERK signal path.

In conclusion, the present research unveiled that gemcitabine combined with XCT790 synergistically inhibited cell viability, proliferation, migration, invasion, EMT and tumor growth via triggering G0/G1 celluler cycle retardation and apoptotic activity via the suppression of ERRα and MEK/ERK pathway in PC. These observations displayed that the combination of gemcitabine and XCT790 might be a novel and potential treatment regimen for the therapy of PC.

## Supplementary Material

Supplementary figures.

## Figures and Tables

**Table 1:** GI_50_ and treatment doses of gemcitabine and XCT790 in each cell line.

**Cell lines**	**Gemcitabine GI_50_ (μmol/L)**	**XCT790 GI_50_ (μmol/L)**
PaTu8988	0.61	3.84
PANC1	0.77	8.76
Mia PaCa-2	0.31	3.98
HPNE	0.68	16.41
		
**Gemcitabine + XCT790 doses**	**Gemcitabine (μmol/L)**	**XCT790 GI_50_ (μmol/L)**
PaTu8988	0.60	3.00
PANC1	0.70	8.00
Mia PaCa-2	0.30	3.00
HPNE	0.60	16.00

## Abbreviations

PC: Pancreatic cancer
ERRα: Estrogen-related receptor alpha
PDX: Patient-derived xenograft
EMT: Epithelial-to-mesenchymal transition
FDA: Food and Drug Administration
DMSO: Dimethyl sulfoxide
NC: Negative control
SiRNA: Small interfering RNA
MOI: Multiplicity of infection
OD: Optical density
CI: Combination index
PI: Propidium iodide
IP: Intraperitoneal
IHC: Immunohistochemistry
HBSS: Hank's balanced salt solution
RFU: Relative fluorescence units
SD: Standard deviation
GI_50_: Half maximal growth inhibition concentration
