# Supplementary material for: Gemcitabine and XCT790, an ERRα inverse agonist, display a synergistic anticancer effect in pancreatic cancer
Source: Int J Med Sci. 2022 Jan 4;19(2):286–98. doi: 10.7150/ijms.68404 (PMC8795805; doi:10.7150/ijms.68404)
Supplement: Supplementary file 1 — Supplementary figures. [file ijmsv19p0286s1.pdf]

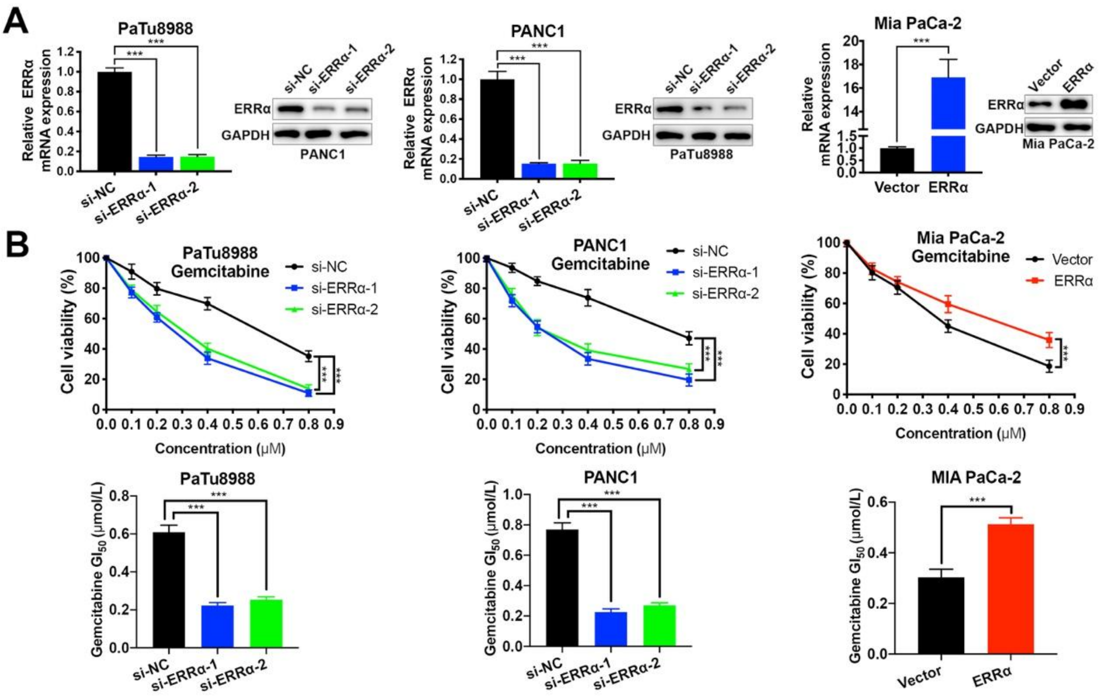

Figure S1. ERRα promoted gemcitabine resistance in PC. A, The expression levels of ERRα in PC cell lines was confirmed at the mRNA and protein level by qRT-PCR and western blot, respectively. B, Cell viability at various concentrations of gemcitabine was measured by CCK-8. The GI50 values of gemcitabine were shown as bar chart. The data were analyzed using Student's t-tests and represented as mean ± SD, ***P < 0.001.

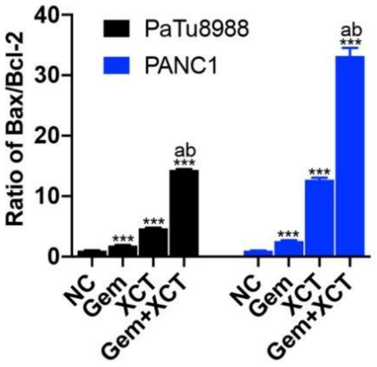

Figure S2. Gemcitabine and XCT790 strongly increased the ratio of Bax/Bcl-2 in PC cells. The expression of Bax and Bcl-2 was analyzed by western blot. Relative protein expression of Bax and Bcl-2 was quantified using ImageJ software and normalized to β-tubulin. The bar chart shows the ratio of Bax/Bcl-2 in PC cells after treatment with gemcitabine and/or XCT790. The data were analyzed using Student's t-tests and shown as the mean ± SD of three independently performed experiments. ****P* < 0.001 compared with the NC group; a and b represent to compare with the GEM or XCT group, *P* < 0.05, respectively.
